# The effect of central bank communication on sovereign bond yields: The case of Hungary

**DOI:** 10.1371/journal.pone.0245515

**Published:** 2021-02-04

**Authors:** Ákos Máté, Miklós Sebők, Tamás Barczikay

**Affiliations:** Centre for Social Sciences, ELKH, Budapest, Hungary; Sam Houston State University, UNITED STATES

## Abstract

In this article we investigate how the public communication of the Hungarian Central Bank’s Monetary Council (MC) affects Hungarian sovereign bond yields. This research ties into the advances made in the financial and political economy literature which rely on extensive textual data and quantitative text analysis tools. While prior research demonstrated that forward guidance, in the form of council meeting minutes or press releases can be used as predictors of rate decisions, we are interested in whether they are able to directly influence asset returns as well. In order to capture the effect of central bank communication, we measure the latent hawkish or dovish sentiment of MC press releases from 2005 to 2019 by applying a sentiment dictionary, a staple in the text mining toolkit. Our results show that central bank forward guidance has an intra-year effect on bond yields. However, the hawkish or dovish sentiment of press releases has no impact on maturities of one year or longer where the policy rate proves to be the most important explanatory variable. Our research also contributes to the literature by applying a specialized dictionary to monetary policy as well as broadening the discussion by analyzing a case from the non-eurozone Central-Eastern region of the European Union.

## Introduction

The volatility of asset prices, spreads and returns is a premier field of study in monetary and financial economics. The key metrics, such as stock returns and bond yields, were traditionally explained by financial asset pricing models and macroeconomic fundamentals [1–4]). More recently, the role of macroeconomic news (both as a dependent and explanatory variable) gained prominence [5, 6]. These studies were rooted in the text as data paradigm which allows for the quantitative analysis of qualitative data, such as textual sources [7, 8].

Sovereign bond yields were also scrutinized in similar research designs [9]. In one example, Altavilla, Giannone, and Modugno [10] analyzed the reaction of the U.S. Treasury bond market to macroeconomic news. In more recent studies the focus has been extended to central bank communication as it carries a ‘forward guidance’ for market participants [11, 12]. The key instruments for forward guidance are interviews, press releases and policy council minutes. These may or may not contain a quantitative interest rate prognosis, therefore the intentions of policy-makers have to be extracted from these sources using quantitative text analysis techniques [13].

However, these text mining techniques are rarely used in a monetary policy context when it comes to Central and Eastern Europe (CEE). In their review, Brzeszczynski, Gajdka and Kutan [14] find that the scarce literature on the CEE region shows that asset prices are vulnerable to external spillover effects from the US monetary policy. Furthermore, there is some evidence for verbal comments by monetary policymakers to have an influence on exchange rates. The closest examples of using textual variables are a few studies on the effect of macroeconomic announcements on composite stock returns or on the effect of central bank communication on media narratives [15, 16]. The CEE cases offer a new perspective as these are small, open economies which are more vulnerable to external shocks and, therefore, may highlight a different dynamic vis-á-vis bigger and less globalized countries.

In this article we fill this gap in the literature by investigating how the public communication of the Hungarian Central Bank’s Monetary Council (MC) affects Hungarian sovereign bond yields. This research ties into the advances made in the financial and political economy literature which rely on extensive textual data and quantitative text analysis tools. While prior research demonstrated that forward guidance, such as council meeting minutes can be used as predictors of rate decisions, we are interested in whether they are able to directly influence asset returns as well. In order to capture the effect of central bank communication, we measure the latent hawkish or dovish sentiment of MC press releases from 2005 to 2019 by applying a sentiment dictionary, a staple in the text mining toolkit.

We estimate the effects of these documents on bond yields by analyzing the common stochastic trends between the sentiment of MC press releases, interest rate decisions and bond yields using autoregressive-distributed lag models. Our results show that central bank talk is not cheap when it comes to sovereign debt in Hungary—but only in regard to intra-year bond yields. Longer term yields are only cointegrated—i.e.: have a long-run equilibrium—with the policy rate. These results point to the potential importance of context and the varying effectiveness of very similar signaling techniques across domains and time as—at least in some research for Western European countries—forward guidance has a more significant impact further down the yield curve. We offer a three-fold contribution to the literature. First, we apply a quantitative text analysis framework which utilizes a dictionary tailored to the domain of monetary policy (as opposed to working with general purpose, or even financial, keywords). Second, we broaden the discussion by analyzing a non-eurozone case from the Central-Eastern region of the European Union. Third, and most importantly from a substantive perspective, we offer fresh insights with regards to the dynamics related to effectiveness of central bank communication.

In what follows we first provide an overview of relevant literature. Next, we put forth our theoretical framework. We proceed with an introduction of our empirical strategy and methods. The analysis section offers regression results related to our research question. The final section concludes.

## The effectiveness of forward guidance in shaping the yield curve

Most central banks in developed countries implement monetary policy by “controlling a short-term nominal rate of interest” [17, p.213]. In basic models, the short-term rate is linked to inflation and output through some sort of the Taylor-rule. The equation determines the nominal interest rate as a reaction function of inflation and the output gap. Empirical studies have largely confirmed that U.S. monetary policy in fact followed some sort of a Taylor-rule for an extended period of time, although more recent studies estimating policy rules for the more general sample of OECD countries have shown a deviation from at least since the early 2000s from this concept [18, 19, p.383]. In these models the monetary policy transmission mechanism starts with the setting of the nominal interest rate and works its way through the economy through money market rates, expectations and, in the next step, through bank rates, exchange rates and asset prices. Forward bond yields are defined on the market based on expectations related to the short rate and a term premium related to the maturity of the security. This expectation theory of the term structure is the baseline model for analyzing the yield curve.

The yield curve is the representation of a function of the return of bonds with different maturities. In other words, it represents the term structure of interest rates over time. The central bank can directly influence the level of short maturity yields (such as overnight rates) through the use of open market operations [20, p.209]. However, the terms of bonds are interconnected as the entire curve is adjusted to short term fluctuations through trading. Furthermore, long yields are also moved by expectations of the inflation rate and the anticipations related to the reaction of the monetary authority to the aforementioned inflation expectations [17, p.457]. These anticipations are mainly derived from central bank communication in relation to macroeconomic trends and future target rate changes. Such ‘forward guidance’ is certainly not a new phenomenon, but it gained prominence in the toolkit of central bankers during and after the financial crisis of 2007-2009 [21]. Furthermore, with the onset of a Japanese-style deflationary environment moving policy rates closer to the zero lower bound in many developed economies it became ever more relevant as a mechanism for conducting monetary policy [17, p.32].

Forward guidance can be implemented on different levels of abstraction and with varying degrees of commitment. According to Barwell and Chadha [22, p.51] two major types of forward guidance are the revelatory and confirmatory forms of communication. The former presents new information about a change in the reaction function while the latter re-affirms the (timeless) central bank’s reaction function in extraordinary times. Since guidance cannot be offered unconditionally, according to Svensson [23] the best way to implement forward guidance is to set a specific path for interest rates which will be evaluated by market participants in terms of its escape clauses, economic developments and uncertainty.

In their review of the practice of forward guidance by major central banks Csortos, Lehmann, and Szalai distinguish three levels of policy implementation [24, p.51]. In its most abstract form, it refers to general tools of transparency, such as the publication of inflation reports and council minutes. On the intermediate level of predictions, the central bank communicates its conditional projection for the future path of interest rates. In its most concrete form, forward guidance initiates a commitment on behalf of the monetary authority. This may be time-contingent (“for the foreseeable future”), or state-contingent (“after the asset purchase program ends”), and both come in open-ended or specified form (“this policy will be in effect through May 2020”). MNB practice—as per its “future strategic framework (…) for unconventional monetary policy instruments [1]”—point to the adoption of the looser definition [25]: “In its forward guidance, the Magyar Nemzeti Bank indicated the long-term maintenance of loose monetary conditions, and thereby it successfully guided the expectations of economic agents.” In this paper we also include in the concept of forward guidance any official communication which may serve as the basis of future monetary policy decisions, regardless of whether the policy rate or the interest rate path is mentioned or not.”

The effectiveness of forward guidance has shown varied results for different jurisdictions, time frames, and also with regards to the abovementioned various forms of implementation (including quantitative and qualitative guidance). In their cross-country survey, Ehrmann, Gaballo, Hoffmann, and Strasser [26] report that forward guidance “mutes the response to macroeconomic news in general, but that calendar-based forward guidance with a short horizon counterintuitively raises it.” They also show that uncertainty is only reduced when the increase in precision of public information is sufficiently large. However, despite attempts at forward guidance, in the cases of small open economies, such as Hungary, the external environment can dominate the communication of central bank officials. A brief case study of the Hungarian National Bank shows such a scenario when bond yield movements were a result of international factors. [27] There are evidence using central bank communications in the CEE region that forward guidance and increased transparency results in decreased forecast dispersion from private forecasters. Notably Jain and Sutherland [28] finds that forward guidance (time and state-contingent) increases private forecast consensus on upcoming rate changes. Furthermore in case of Poland research indicates that after the National Bank of Poland (NBP) implemented a forward guidance policy it resulted in decreased forecasts dispersion (even though this period also contained severe image crises periods for the NBP) [29, 30]

Charbonneau and Rennison [31] assess the effectiveness of forward guidance in the practice of six central banks. They also differentiate three types of forward guidance (qualitative, time contingent and state contingent). Their summary of extant literature shows that forward guidance is “effective in (1) lowering expectations of the future path of policy rates, (2) improving the predictability of short-term yields over the near term and (3) changing the sensitivity of financial variables to economic news.” Andersson and Hofmann [32] investigated three inflation targeting countries. Their results show that while the publication of a quantitative guidance in the form of an own interest rate path contributed to anchoring long-term inflation expectations, only the central bank of New Zealand was able to maintain a leverage on the medium-term structure of interest rates.

Detmers, Karagedikli, and Moessner [33] also investigate the monetary policy of New Zealand, but this time by comparing the effects of quantitative (interest rate forecast) and qualitative (in the form of a macroeconomic outlook) forward guidance. Their findings show that “announcements that include an interest rate forecast lead to very similar market reactions across the yield curve as announcements that only include written statements.” Their interpretation of these results points to the relative insignificance of the exact form of communication.

In a similar study for the FED, Moessner [34] presents results which indicate that “open-ended and time-contingent forward guidance announcements led to a significant reduction in forward US Treasury yields at a wide range of horizons”. Interestingly, the largest reduction occurred at the 5-year ahead horizon for both kinds of announcements. Nevertheless, the results were ambiguous in that state-contingency based forward guidance led to a significant increase in forward US Treasury yields for horizons of 3–7 years ahead. This effect, however, may have also be due to asset purchase announcements made in the same statements.

The findings of Gürkaynak et al. [6, p.425] related to the effect of macroeconomic news on U.S. bond yields seemingly contradict standard macroeconomic models and the abovementioned findings. The baseline scenario would suggest that short-term interest rates “return relatively quickly to a deterministic steady state after a macroeconomic or monetary policy shock” and “one would expect virtually no reaction of far-ahead forward rates to such shocks”. Their evidence suggests that forward rates at long horizons, in fact “react significantly to a variety of macroeconomic and monetary policy surprises”. However, it is notable that their main explanatory variables are not monetary policy announcements or target rate decisions, but data on federal funds futures rates and deviations of macroeconomic data from the market consensus.

Brand, Buncic, and Turunen [35, 1269] focus on the euro area money market yield curve on dates when the ECB regularly sets and communicates decisions on policy interest rates. They show that “ECB communication during the press conference may result in significant changes in market expectations of the path of monetary policy” and that “these changes have a significant and sizeable impact on medium to long-term interest rates.” In a similar study for the ECB, Hubert and Labondance (2018) found that announcements lowered the term structure of private short-term interest rates. This finding is corroborated for U.S. data by Evans and Marshall [36] who describe monetary policy shocks (in the form of, inter alia, the Federal Funds Rate and total reserves) which have a short-lived, 6 months to one year, effect on the yield curve.

Goodhart [37, p.153] is more skeptical regarding the merit of forward guidance. He indicates that in Sweden and Norway (up to 2010) “official path adjusts to market rates, rather than vice versa, except on short horizons in Sweden where there exists a two-way relationship.” He also notes that time-consistent guidance in Canada in 2009 and macroeconomic state-contingent in the UK in 2013 “have been largely successful in influencing the short end of the yield curve but have had no effect at longer horizons”.

Based on these empirical results in this paper we investigate four hypotheses:

Hypothesis 1: The effect of forward guidance is not independent of policy (rate) decisions

H1 posits a clear coherence between various instruments of monetary policy, namely short-term interest rate targets and forward guidance. Except for extreme cases related to the effective lower bound, this is a plausible supposition (to the point that central bank communication may be successfully used to predict rate changes) [38, 39].

Hypothesis 2: Forward guidance is not effective for money market yields

H2 further refines H1 in that its states that securities with an intra-year maturity will have priced in imminent rate changes and forward guidance on future short-term interest rates offers no additional information over these changes effectively guided by the interest rate corridor of official lending and deposit rates.

Hypothesis 3: Forward guidance is effective for short to long term bonds of a maturity of one year or longer

H3 describes the key tenet of forward guidance by claiming that it is effective in flattening the yield curve.

Hypothesis 4: The effect of forward guidance declines over the yield curve

**Fig 1:**
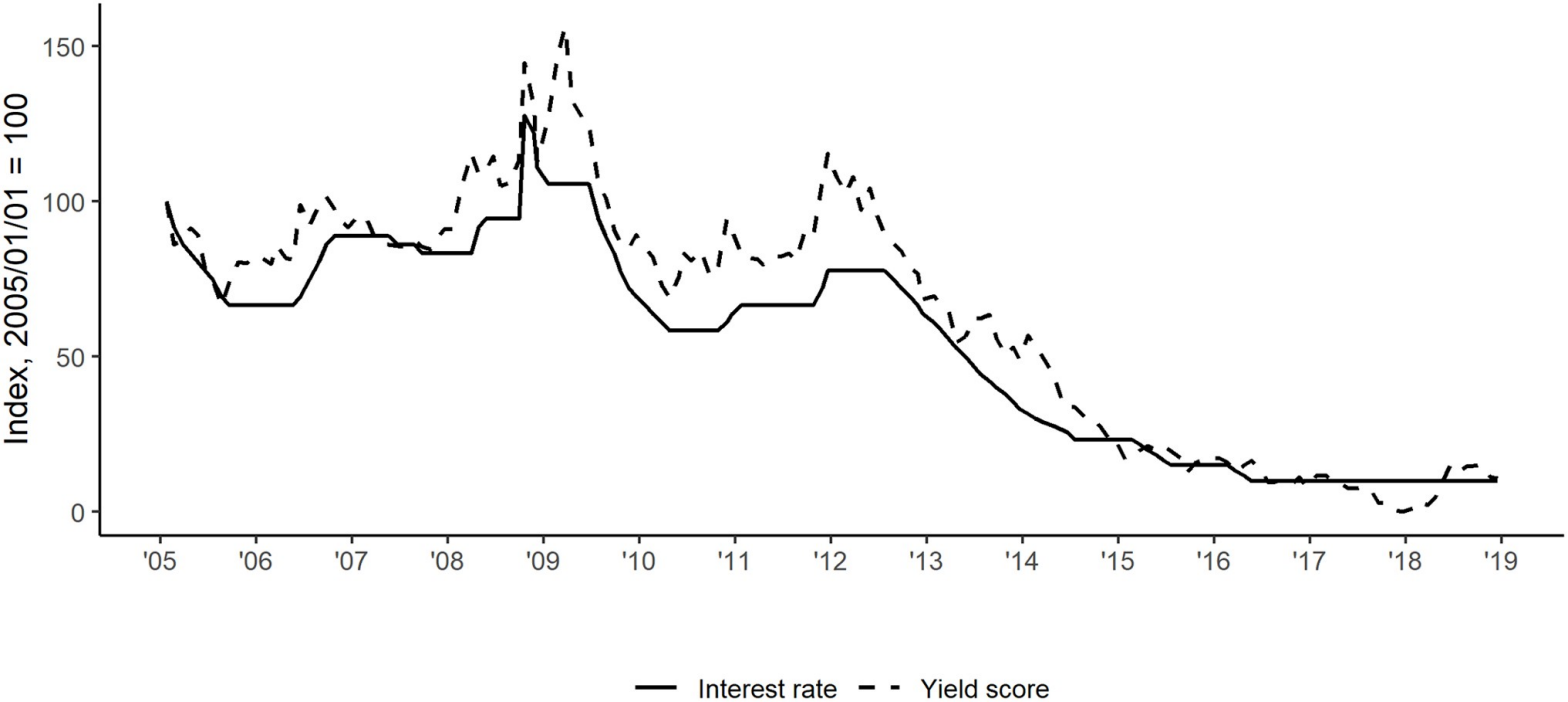
Co-movement of the interest rate and the yield factor score.

H4 refers to macroeconomic uncertainties and various risk factors presented in Fig 1, as well as credibility failures on behalf of the central bank.

## Measuring the effect of monetary policy sentiment on bond yields

The volatility of asset prices, spreads and returns is a premier field of study in monetary and financial economics. Traditionally, fluctuations in stock returns and bond yields, as well as those related to commodity prices, were explained by asset pricing models, the analysis of supply and demand in asset markets, the productivity and profitability of the company in question, technical analysis as well as macroeconomic fundamentals [1–4, 40]. More recently, other factors, mainly those used in political economy models which retain a wider cast in terms relevant variables vis-á-vis traditional finance, were included in order to better account for real-life processes. One such major area of improvement pertains to the effect of macroeconomic news, as well as the verbal and written communication of official and market actors which are operationalized in the context of the text as data paradigm [7, 8].

The main textual input source used in these studies is business and macroeconomic news [6]. Birz and Lott Jr [5] applied a headline classification scheme in order to use news coverage as an explanatory variable. Their findings indicated that news on GDP and unemployment did, in fact, affect stock returns. In a similar study Boyd, Hu, and Jagannathan [41] found that on average an announcement of rising unemployment is good news for stocks during economic expansions and bad news during economic contractions. Using CDS spread and government bond yield spread as their dependent variable, Fulop and Kocsis [42] use an elaborate sentiment coding scheme to estimate the effects of local and global news coverage of various issues. In line with the literature they find that both the CDS and the bond yield spreads are affected by the news coverage.

An ever-growing sub-branch of the literature focuses on understanding monetary policy proper within the text as data paradigm. Baerg and Lowe [43] estimate a textual Taylor-rule by using a topic-based text analysis and scaling methods to gauge the preferences of FED decision-makers. Sovereign bond yields were also scrutinized in research designs similar to those applied for stock prices as a dependent variable. Altavilla et al. [10] analyzed the reaction of the U.S. Treasury bond market to macroeconomic news. They found that macroeconomic news explained about one-third of the low frequency (quarterly) fluctuations in long-term bond yields. Bond market volatility serves as the dependent variable for a slew of other media-based studies, which sometimes analyze results from the standpoint of monetary policy [9, 11, 12].

The key monetary policy instruments for disseminating forward guidance are interviews, press releases and policy council minutes. These may or may not contain a quantitative interest rate prognosis, therefore the intentions of policy-makers have to extracted from these sources using qualitative or quantitative text analysis techniques [13]. One key approach is sentiment analysis, which in extant studies has been mostly implemented with a dictionary-based technique. This refers to a quantitative text analysis method which is widely applied in both political science and in economics and finance [7, 8, 44]. To get the sentiment score for a given document in the corpus we have to use a specific dictionary that represent the same sentiment (e.g.: hawkish or dovish) and then measure the relative frequency of these words in the corpus.

An important caveat for this technique concerns the validity of the applied dictionary. In their seminal work Loughran and McDonald [45] show that general purpose sentiment dictionaries cannot be used to assess the sentiment of economic texts because due to misclassification problems (e.g.: the word “vice” in a finance or economic text is more likely to be related to the corporate position of vice-president than to any negative connotation).

Apel and Blix Grimaldi [46] constructed two quantitative measures—dove and hawk—by an automated search on a set of monetary policy meetings at the Riksbank, Sweden’s central bank. They applied a context-specific list which consisted of combinations of a noun and an adjective such as “higher inflation” and “lower growth”. In the next step they looked for adjective pairs with “inflation”, “cyclical position”, “growth”, “price”, “wages”, “oil price” and “development”, a list of words which they posited to reflect the goals of policymakers. In a subsequent study they extend their dictionary in order to better contextualize economic keywords to measure the difference between the minutes and transcripts of the Federal Reserve’s FOMC [47].

In a similar manner, Picault and Renault [48] developed a field-specific dictionary to measure the dovish, neutral, or hawkish stance of the European Central Bank (ECB) based on its press conferences. They used term-weighting and contiguous sequence of words (n-grams) with an aim to better capture the subtlety of central bank communication.

Despite their ongoing application to the U.S., Western Europe, and even India or South Korea, the aforementioned text mining techniques are rarely used in a monetary policy context when it comes to Central and Eastern Europe [49, 50]. The closest examples of using textual variables are a few studies on the effect of macroeconomic announcements on composite stock returns or on the effect of central bank communication on media narratives [15, 16]. In light of these considerations, we follow the literature on the European Central Bank (ECB) when it comes to the operationalization of qualitative forward guidance.

## Data and methods

### Dependent variable

In our models, intra-year, short term and long-term government bond yields serve as the dependent variables. There is some ambiguity in the literature and official documents as to what constitutes short or long term maturity for government bonds. McCauley and Remolona [51] consider government securities with a tenor of more than one year to be long term. Contrastingly, a member of the executive board of the ECB used a collection of 5, 10, 15 and 20 year maturities for analyzing the long end of the yield curve. The U.S. Department of Treasury refers to “long term rates” by listing securities of a maturity of longer than ten years.

In our reference country of Hungary, ‘Government Bonds’ are considered to be long term investments. They are interest-bearing securities with a maturity longer than one year. Currently, they are issued with tenors of 3, 5, 10 and 15 years. As a further complication, retail banks often consider papers of 3 or 5 years of maturity to be medium-term investments while some short-term bond funds follow a 1 to 3-year index of securities. In order to evaluate the effect of press releases on different horizons of the term structure, we created three distinct dependent variables out of the official Hungarian bond yield data sources using principle component analysis. Technically, intra-year yields (Yield_intra year_) were constructed from bonds with maturity rates of three and six months, while bonds exceeding six months were grouped into two categories. The other short-term category (Yield_1-3y_) refers to bonds with a maturity rate of one and three years, and long-term yields (Yield_long term_) combine five, ten and fifteen-year bonds. As expected, the assembled factor scores are highly correlated with the individual returns.

**Fig 2:**
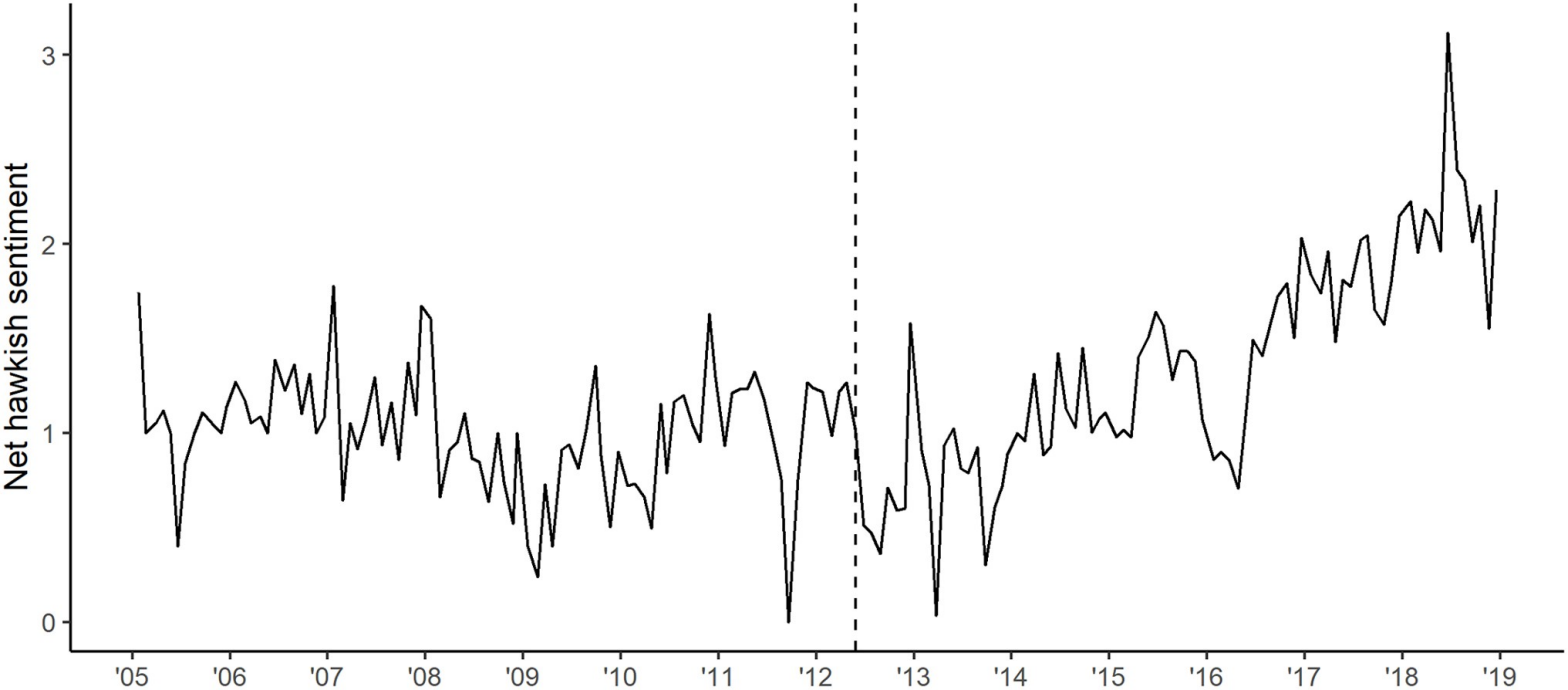
The sentiment time series with the structural break.

In the 2010s Hungarian monetary policy produced a remarkable change of course vis-á-vis the preceding decades. From the end of 2008, interest rates decreased from the peak of 11.50 percentage points to 0.90 and did not change since April of 2016. Fig 2 shows that that yield factor scores followed suit. The co-movement of these time series indicate a strong correspondence between the two variables (which is reinforced by the fact that their Pearson’s r equals 97.39, 97.95 and 92.50 respectively as government bond maturity increases).

What is interesting, and telling of cheap money, deflationary environment of late 2010’s global monetary policy, is that a generally dovish sentiment was (curiously, yet reasonably) accompanied by low interest rates and yields (see Fig 1). Here, the effects of the historic rate cutting campaign of the accommodating Matolcsy governorship is also evident, which adds face validity to our measurement strategy.

### Explanatory variable: The sentiment of central bank press releases

In our models, the sentiment of central bank communication is a key explanatory variable of interest. To measure the sentiment of the central bank press releases we created a monetary sentiment dictionary that improves upon the general sentiment dictionaries (such as the Harvard IV-4) and even finance specific ones such as the Loughran and McDonald [45], Apel-Grimaldi [46] or the most recent, Apel, Grimaldi and Hull [47]. We provide more information on and results for alternative operationalizations of the textual variable in the Appendix B and D.

The monetary sentiment variable is constructed from the corpus of the official, English language press releases of the Monetary Council (MC) of Magyar Nemzeti Bank (MNB), the central bank of Hungary. The press releases are distributed alongside the rate decision of the MC and they provide a brief explanation into the deliberations of the council. The corpus contains all of the published press releases between 2005 and 2018. As the summary table of word counts shows (see Table 1) the length of these increased over the past decade, from 360 mean word count in 2005 to 1234 in 2018. Nevertheless, our expectation is that the net sentiment of these documents is unaffected by this increase in length since, in order to account for the changes of press release verbosity over time, we applied relative term frequency weights during the sentiment score calculations.

**Table 1:** Descriptive statistics of the textual variable in word count.

year	N	Mean	S.d	Min	Max
2005	12	377.67	230.12	86	796
2006	12	362.25	186.31	67	713
2007	12	405.50	111.33	249	670
2008	12	359.08	232.31	21	955
2009	12	510.75	117.69	327	774
2010	12	502.92	83.75	363	672
2011	12	681.75	113.64	494	894
2012	12	849.58	478.57	515	1955
2013	12	925.08	507.20	486	1947
2014	12	882.83	315.62	591	1437
2015	12	886.00	278.71	587	1485
2016	12	833.25	75.67	734	973
2017	12	1146.58	106.30	991	1333
2018	12	1299.00	188.28	1104	1848
2019	12	1160.08	171.96	821	1336

Our new dictionary is built from three main components. Firstly, we selected the relevant macroeconomic key terms from the widely used macroeconomics textbook of Hall and Taylor [52] and examined the context of these keywords in our corpus. Secondly, following the extant literature, we compiled a list of valence shifters which alter the sentiment attached to a keyword [45, 46]. This was necessary to correctly measure sentiment in a monetary and macroeconomic policy context. Thirdly, we classified each macroeconomic term—valence shifter combination (e.g.: increase + growth) as hawkish or dovish. We validated these combinations with three independent annotators, and we kept the pairs which received a majority of votes.

Out of our macroeconomic terms we created five categories based on how they behave with valence shifters. In each category each term behaves the same when the same valence shifter is applied. These “normal terms” are the largest group and they behave intuitively: positive valence shifters are hawkish (as these are usually pointing towards an inflationary pressure) and negatives are dovish. We classified *inflation* (and related terms), *deficit* (and related terms), *unemployment* and *interest* as special cases where a more granular approach was required. Each term has a corresponding hawkish and dovish valence shifter list. The sentiment scores are obtained from a relative frequency weighted document-feature matrix (which represents the occurrences of words from the entire lexicon of the corpus in each constituting document) where our features are sentences. This means that it is unlikely that the macroeconomic term and a particular valence shifter that alters the sentiment are not related. Using sentences as tokens also guards against false positives which could occur if sentence limits are not taken into account. We score each document on hawkish and dovish scales. For the hawkish measure we sum up the relative word frequencies of regular and irregular macroeconomic term and valance shifter combinations and vice versa for the dovish scale. Finally, we construct a net hawkishness index for each document *d*.

$$\begin{array}{c} {\text{nethawkish}}_{d}=\left({\text{hawkish}}_{d}-{\text{dovish}}_{d}\right)+1 \end{array}$$ (1)

where *hawkish*_*d*_ for document *d* is composed of the sum of the relative frequency of regular and irregular macroeconomic terms and their valence shifter combinations. The *dovish*_*d*_ for document *d* is constructed in the same way. We add the value of one to each in order to avoid negative numbers.

This more granular approach improves upon the widely used Loughran-McDonald and the Apel, Grimaldi and Hill dictionaries. In case of the Loughran-McDonald we demonstrate this with the following sentence that comes from the press release of October 30 in 2012: ‘[…] the *unemployment* rate was close to its historically *low* level.’ The Loughran-McDonald dictionary would give it a negative score because low is classified as negative. Similarly, general sentiment dictionaries, such as the Harvard IV-4, would also classify it as negative for the same reason. However, our approach correctly identifies this as a hawkish sentence, as unemployment is an irregular term and it has word low (which is in the hawkish valence shifter list for irregular terms) in its 5-word window (another element of our approach which accounts for real-life syntax). This way our monetary sentiment dictionary correctly gives the example sentence the score of ‘hawkish’. Compared to the Apel, Grimaldi and Hill dictionary we use a broader set of economic terms which allows for a more fine grained scoring of documents. The following sentence is from the press release of September 20 in 2011: ‘Large individual investment projects implemented in manufacturing will only partly offset *weak investment* activity.’ The AGH dictionary does not contain the term ‘investment’ and thus would miss this sentence. The monetary dictionary described in Table 2 would correctly score this sentence as dovish.

**Table 2:** The composition of the macroeconomic sentiment dictionary.

List type	Dictionary items
terms_normal	business activity, capital flow, consumption, demand, disinflation, economic activity, economic growth, economic upturn, employment, exchange rate, export, gdp, investment, loans, output, wage, wages
hawk_normal	buoyant, fast, faster, fastest, favourable, grew, grow, growing, grown, growth, high, higher, highest, improve, improved, improvement, improving, increase, increased, increasing, picked up, picking up, rise, risen, rising, rose, stabilise, stabilised, stable, stronger, strong, strongest, upwards
dove_normal	decline, declined, declining, decrease, decreased, decreasing, deteriorate, deteriorated, deteriorating, deterioration, downwards, fall, fallen, falling, fell, low, lower, lowest, shrink, slow, slowdown, slowed, slower, slowest, slowing, subdue*, subdued, unfavourable, weak, weakened, weakening, weaker, weakest, worse, worsen
deficit_term	budget deficit, government deficit, government balance
deficit_dove	decline, declined, declining, decrease, decreased, decreasing, downwards, fall, fallen, falling, fell, improve, improved, improvement, improving, low, lower, lowest, shrink, slow, slowdown, slowed, slower, slowest, slowing, subdue*, subdued
deficit_hawk	deteriorate, deteriorated, deteriorating, deterioration, favourable, grew, grow, growing, grown, growth, high, higher, highest, increase, increased, increasing, picked up, picking up, rise, risen, rising, rose, stabilise, stabilised, stable, stronger, strong, strongest, unfavourable, upwards, weak, weakened, weakening, weaker, weakest, worse, worsen
inflation_term	consumer price index, cpi, inflation, inflationary, price, prices
inflation_dove	decline, declined, declining, decrease, decreased, decreasing, downwards, fall, fallen, falling, fell, improve, improved, improvement, improving, low, lower, lowest, shrink, slow, slowdown, slowed, slower, slowest, slowing, stabilise, stabilised, stable, subdue*, subdued, surplus, weak, weakened, weakening, weaker, weakest
inflation_hawk	deteriorate, deteriorated, deteriorating, deterioration, fast, faster, fastest, favourable, grew, grow, growing, grown, growth, high, higher, highest, increase, increased, increasing, picked up, picking up, rise, risen, rising, rose, stronger, strong, strongest, unfavourable, upwards, worse, worsen
unemp_term	unemployment
unemp_dove	deteriorate, deteriorated, deteriorating, deterioration, favourable, grew, grow, growing, grown, growth, high, higher, highest, increase, increased, increasing, picked up, picking up, rise, risen, rising, rose, stronger, strong, strongest, unfavourable, upwards, worse, worsen
unemp_hawk	decline, declined, declining, decrease, decreased, decreasing, downwards, fall, fallen, falling, fell, improve, improved, improvement, improving, low, lower, lowest, shrink, slow, slowdown, slowed, slower, slowest, slowing, stabilise, stabilised, stable, subdue*, subdued, weak, weakened, weakening, weaker, weakest
interest_term	interest rate
interest_dove	decline, declined, declining, decrease, decreased, decreasing, fall, fallen, falling, fell, low, lower, lowest
interest_hawk	favourable, grow, high, higher, highest, increase, increased, increasing, rise, risen, rising, rose

### Modeling strategy

To test our hypotheses we use autoregressive-distributed lag (ARDL) models estimated with ordinary least squares method. This approach allows us to identify the potential cointegrating relationships among our time series (the sentiment, yield scores and interest rate) which, if not detected, could lead to spurious regression problems due to model misspecification. Using ARDL models overcomes the problems of the biased estimates and standard errors of a more traditional OLS model which might falsely identify association between variables as statistically significant (or insignificant). Moreover, compared to the vector error correction models (VECM) our approach has two distinct advantages. Firstly, we estimate a single equation and apply the widely used Pesaran test for cointegrated time series that is able to work with smaller samples [53]. Secondly, a main limitation of the VECM approach is that it cannot be used effectively when one or more of the time series are stationary in the dynamic system.

We estimate the ARDL model in unrestricted error correction model (UECM) form in Eq 2.

$$\begin{array}{c} \Delta y_{t}=c_{0}+\pi_{y}y_{t-1}+\sum_{j=1}^{k}\pi_{j}\Delta x_{j,t-1}+\sum_{i=1}^{p-1}\psi_{y,i}\Delta y_{t-i}+\sum_{j=1}^{k}\sum_{l=1}^{q_{j}-1}\psi_{j,l}\Delta x_{j,t-l}+\sum_{j=1}^{k}\omega_{j}\Delta x_{j,t}+\epsilon_{t} \end{array}$$ (2)

Our dependent variable is differenced in align with the UECM form of the ARDL model. On the right hand side in Eq 2 *c*_0_ indicates the intercept. The second term gives the one period lagged effect of the dependent variable. The third term adds the effect of the one period lagged independent variables. The above mentioned terms are the elements of the error correction part of the model and jointly incorporate the long run effects into the model. The fourth and fifth terms include the effects of the lagged differenced dependent and independent variables. Here *p* denotes the lag order of the differenced dependent variable, such as *q*_*j*_ which is the lag order of the *j*^*t*^ *h* independent variable. The sixth term indicates the contemporaneous effect of the change in the independent variables. In all cases we used the AIC indicator to choose between lag structures of *p* and *q*_*j*_. (In cases where the best model suggested a version where one of our explanatory variables were missing we switched to the next best that incorporated all of them.) In all cases the *k* upper limit denotes the number of explanatory variables that equals with two in all of our models. Terms from four to six are responsible for the short run dynamics of the models. Lastly the *ϵ*_*t*_ term stands for the stochastic error term. Within the ARDL framework, assuming a fully specified model, the error term is normally distributed and free of autocorrelation.

We are estimating three ARDL models, one for each period. The two independent variables in the model are interest rate and the sentiment score of the press releases.

## Results

Before the modelling phase we identified the integration order of our time series using augmented Dickey-Fuller (ADF) and Phillips-Perron (PP) tests. Since the behavior of the nethawkish time series visibly change approximately at the middle of the sample, we applied a constant and time trend augmented Zivot-Andrews test (test statistics: -8.73, 1% critical value: -5.57), and verified our suspicion.

According to the Zivot-Andrews test, there is a structural break at period 89 (May 2012) which can be seen on Fig 2. The variable behaves as a stationary time series before the break, but tends to increase around a sharp trend after the specified date. In order to account for this potentially distorting effect, we tested all our time series for stationarity on three different sample sizes. The first sample contains all periods ranging from 1 to 168, while the second runs from period 1 to 89. Period 90 serves as a boundary and therefore the third sample includes observations from period 90 to 168.

Table 3 contains the results of the estimated stationarity tests. On Panel A and B we represent estimation results that utilize the drift augmented specification of all the tests in question. On Panel C we also included a trend term, to take into account the trending nature of our data. Nethawkish is of special interest to us. When examining the level series, we cannot reject the null hypothesis of a unit root process on a 1% significance level. (This is jointly true for the ADF and PP tests, irrespective of the sample in question.) In contrast, in the case of the shorter subsamples, the null can be rejected on a 5% level according to the ADF test results. It worth’s noting that the subsamples in question are relatively short compared to the whole data series, consisting of 89 and 79 observations in order. Therefore, we accept the more permissive 5% significance level and treat nethawkish as a stationary variable (i.e. we reject the null of non-stationarity) when running regressions on the subsamples. Furthermore, we can see that all remaining time series are non-stationary in their level forms, but become mean reverting after differencing them. This statement is supported by both stationarity tests, on all subsamples.

**Table 3:** ADF and PP stationarity test statistics.

	ADF					PP				
	Level	Difference	1%	5%	10%	Level	Difference	1%	5%	10%
A: Full sample with drift (1-168)										
Yield_long term_	-1.06	-10.03				-1.10	-13.48			
Yield_1-3y_	-0.71	-5.44				-0.84	-12.11			
Yield_intra year_	-0.63	-4.59	-3.46	-2.88	-2.57	-0.71	-11.47	-3.47	-2.88	-2.58
i	-0.79	-4.42				-0.94	-10.17			
nethawkish	-1.71	-12.84				-3.71	-21.25			
B: Subsample with drift (1-89)										
Yield_long term_	-2.46	-7.47				-2.44	-9.70			
Yield_1-3y_	-2.14	-8.36				-2.23	-8.86			
Yield_intra year_	-2.06	-3.35	-3.51	-2.89	-2.58	-1.95	-8.42	-3.51	-2.89	-2.58
i	-1.73	-5.95				-2.00	-7.58			
nethawkish	-2.94	-9.87				-6.76	-16.80			
C: Subsample with trend specification (90-168)										
Yield_long term_	-1.57	-5.89				-1.96	-9.40			
Yield_1-3y_	-0.70	-6.32				-2.09	-8.66			
Yield_intra year_	-2.4	-4.74	-4.04	-3.45	-3.15	-2.35	-8.90	-4.08	-3.47	-3.16
i	-2.68	-3.67				-2.79	-5.30			
nethawkish	-3.62	-4.94				-5.42	-12.97			

Due to the experienced structural break, we estimated all of our dependent variables—Yield_long term_, Yield_1-3y_ and Yield_intra year_—on all three time horizons. Regarding the low number of observations in subsample 2 and 3 we applied separate ARDL models to capture the effect of potential cointegration. Although VECM models could serve as an alternative to ARDL models, a single equation model consumes less degree of freedom and serves our purposes better. Estimation results can be found in Table 4. First and foremost, it is worthy to examine the post-estimation tests of the nine models. All models pass the Breusch-Pagan LM test for autocorrelation in the residuals, thus we cannot reject the null hypothesis of no autocorrelation on any traditional significance levels. In contrast, most of the models suffer from the non-normality of the residuals. Looking at the Q-Q plots of the residuals, only models 3, 6 and 9 have a near perfect distribution. Q-Q plots in our case compare the residuals of the model to a theoretical normal distribution. If outliers are outside the error bound, the normality of the residuals is questionable. See in the Appendix A for the diagnostic plots. This is highly problematic, because standard statistical tests results can only be evaluated under both of the previously mentioned conditions. Therefore, we can only make valid statistical statements about the enumerated models, that were estimated on the last subsample.

**Table 4:** ARDL model results.

	Long-term yield			1-to-3-year yield			Intra-year yield		
Sample	1-168	1-89	90-168	1-168	1-89	90-168	1-168	1-89	90-168
	Model 1	Model 2	Model 3	Model 4	Model 5	Model 6	Model 7	Model 8	Model 9
Intercept	-0.011	0.124*	-0.023	0.0180	0.023	-0.001	0.009	-0.006	0.008
	(0.020)	(0.054)	(0.019)	(0.016)	(0.044)	(0.012)	(0.009)	(0.023)	0.008
*yield*_*t*−1_	-0.154***	-0.208*	-0.158**	-0.248***	-0.208*	-0.165**	-0.274***	-0.268*	-0.356***
	(0.043)	(0.063)	(0.056)	(0.067)	(0.100)	(0.061)	(0.068)	(0.125)	(0.076)
*i*_*t*−1_	0.012***	0.005	0.005	0.020***	0.014	0.009	0.024***	0.022*	0.028***
	(0.003)	(0.005)	(0.005)	(0.006)	(0.008)	(0.005)	(0.006)	(0.011)	(0.006)
*nethawkish*_*t*−1_	0.008	-0.041	0.0178	-0.014	0.007	-0.001	-0.013*	0.016	-0.015**
	(0.011)	(0.027)	(0.010)	(0.010)	(0.024)	(0.007)	(0.006)	(0.013)	(0.005)
Δ*i*	0.060***	0.059***	-0.175**	0.090***	0.094***	-0.033	0.090***	0.092***	0.029
	(0.011)	(0.014)	(0.054)	(0.008)	(0.011)	(0.032)	(0.005)	(0.007)	(0.024)
Δ*nethawkish*	0.026*	0.007	0.010	0.011	-0.001	0.0001	-0.001	-0.002	-0.006
	(0.013)	(0.024)	(0.013)	(0.010)	(0.018)	(0.008)	(0.006)	(0.010)	(0.006)
Δ*yield*_*t*−1_				0.163	0.100		0.068	-0.012	
				(0.088)	(0.106)		(0.083)	(0.073)	
Δ*yield*_*t*−2_				-0.057	-0.266**			-0.264***	
				(0.081)	(0.090)			(0.065)	
Δ*yield*_*t*−3_				0.157*					
				(0.078)					
Δ*i*_*t*−1_	-0.024*			-0.034**	-0.024		-0.010		
	(0.011)			(0.010)	(0.014)		(0.008)		
Δ*i*_*t*−2_	-0.031**			-0.019			-0.016**		
	(0.011)			(0.010)			(0.005)		
Δ*i*_*t*−3_				-0.004			0.010		
				(0.010)			(0.005)		
Δ*i*_*t*−4_				0.040***			0.021***		
				(0.009)			(0.005)		
Δ*nethawkish*_*t*−1_				0.007	-0.041*		0.003	-0.027**	
				(0.012)	(0.018)		(0.007)	(0.010)	
Δ*nethawkish*_*t*−2_				0.025*			0.015*		
				(0.011)			(0.006)		
Δ*nethawkish*_*t*−3_				0.015					
				(0.010)					
R-squared	0.263	0.265	0.250	0.574	0.577	0.240	0.730	0.769	0.427
adj. R-squared	0.230	0.220	0.198	0.530	0.526	0.187	0.708	0.745	0.387
BP test p-value	0.781	0.452	0.191	0.499	0.307	0.780	0.202	0.099	0.919

As expected in time series models, the lagged value of the dependent variable is significant on a 1% level. It is more interesting, that medium—and long run yields—i.e. Yield_1-3y_ and Yield_long term_—are not affected by neither interest rates, nor the sentiment variable. Conversely, in the short run model with Yield_intra year_ as the dependent variable, both of them are highly significant, and have the expected sign. Lagged interest rate was significant on a 1% level with a 0.028 (0.006) parameter estimate, while nethawkish turned up to be -0.015 (0.005) on the same significance level. (None of these variables were significant on any other model specification of the same subsample.) Among the differenced variables—that are responsible for the short run dynamics of the equations -, we can only find a significant effect of the change of interest rate in the Yield_long term_ equation (model 3). From the perspective of model fit we turn to adjusted *R*^2^. Long—and medium term models approximately explain one-fifth of the variability of the dependent variables. A much higher fit is observed in the case of model 9. Roughly 39% of the variation in Yield_intra year_ is explained by the lagged independent and dependent variables.

After the scrutiny of the parameter estimates, we turned to the testing of cointegration in the selected models. We conducted PSS tests of cointegration [53]. The PSS bounds test separates the positive values of an F-distribution into three intervals with a lower—and an upper critical value. Test statistics that fall below the lower bound indicate that the time series are all I(0) non-stationary processes. On the contrary, if the calculated statistics are higher than the upper bound, the test indicates that all variables are I(1) and cointegrated. Results in between the bounds are ambiguous, and thus not appropriate to decide upon the existence of cointegration in the tested equation. Table 5 summarizes the results of the tested models. It is visible that both models estimated on the last subsample with dependent variables Yield_long term_ and Yield_intra year_ are cointegrated on the 1% significance level. In the case of variable Yield_long term_, the 7.90 test statistic is higher than the 1% critical value of 6.36. The same is true for the Yield_intra year_ model with its calculated 8.04 test statistic. For the last potential model with Yield_1-3y_ as the dependent variable, the test statistic of 5.56 exceeds the 5% upper critical value of 4.85, but falls in between the bound on the 1% significance level. Thus the existence of a cointegrating relationship is less straightforward.

**Table 5:** PSS test results for different dependent variables and subsamples.

	Yield_long term_			Yield_1-3y_			Yield_intra year_			Sig. Level	LB	UB
Sample	1-168	1-89	90-168	1-168	1-89	90-168	1-168	1-89	90-168	1%	5.15	6.36
F-stat.	5.308	3.949	7.905	4.627	1.906	5.562	5.589	2.590	8.045	5%	3.79	4.85

It is still questionable whether all the variables in the selected models are mutually cointegrated, or there is only pairwise cointegration among the yield curves and the interest rate, excluding the sentiment variable from the long run relationship. The parameter estimates shown that the nethawkish sentiment variable was only significant in the short run yield equation in model 9 which makes this claim worthy of scrutiny. To test this possibility, we have to return to the stationarity tests of Table 3. We assumed that the nethawkish variable is non-stationary on a 1% significance level, but it can be shown that testing for 5% results in rejecting the null of an unit root process. The test statistic of the ADF test was -3.62 which is higher than the -4.04 1% critical value. (Moreover, we must keep in mind that the PP test clearly rejected the null).

We continued with conducting Johansen trace tests for cointegration, which test perform less accurately when I(0) variables are present [54] (See the results in the Appendix). Keeping this in mind we interpreted our findings with caution. We estimated the test for all yield horizons in three specifications. In the firs we included all three time series. The second specification was motivated by the literature, thus we included the yield curves together with the interest rate (i.e. excluding the sentiment variable). The third version was the mirror image of the previous. We left out interest rate for nethawkish. Results show that in case of the medium—and long run yields, Yield_1-3y_ and Yield_long term_ are cointegrated with the interest rate in specification 2, but not cointegrated with nethawkish in specification 3. In both cases the combined version containing all three variables show that only two of them are cointegrated meaning that this relationship can only occur between yields and interest rates. The case of Yield_intra year_ was different. Both pairwise specifications indicated that there is a cointegrating relationship. The series of tests in the first specification in turn shown that there are two cointegrating vectors indicating that all three time series are mutually cointegrated.

The null hypothesis of zero cointegrating relationship (*r* = 0) was rejected on a 1% significance level (test statistic: 70.73 > 1% critical value: 37.22), such as the next level of the test with the null of *r* < = 1 (test statistic: 26.37 > 1% critical value: 23.52). In the last step we could not reject the null hypothesis of two or less cointegrating vector on a 10% significance level because the test statistic of 3.84 could not surpass the critical value of 6.50. Because all previous levels in the line of trace tests were rejected, meaning increasingly higher number of cointegrating vectors, the null hypothesis must be *r* = 2 instead of *r* < = 2, thus there is exactly two cointegrating relationship. This means that all three time series are mutually cointegrated and have a common stochastic trend. Based on this evidence we believe that medium—and long term yields are not affected by the sentiment scores of central bank communication, thus we continue with examining the shock responses of interest rates and sentiment shocks in model 9.

**Fig 3:**
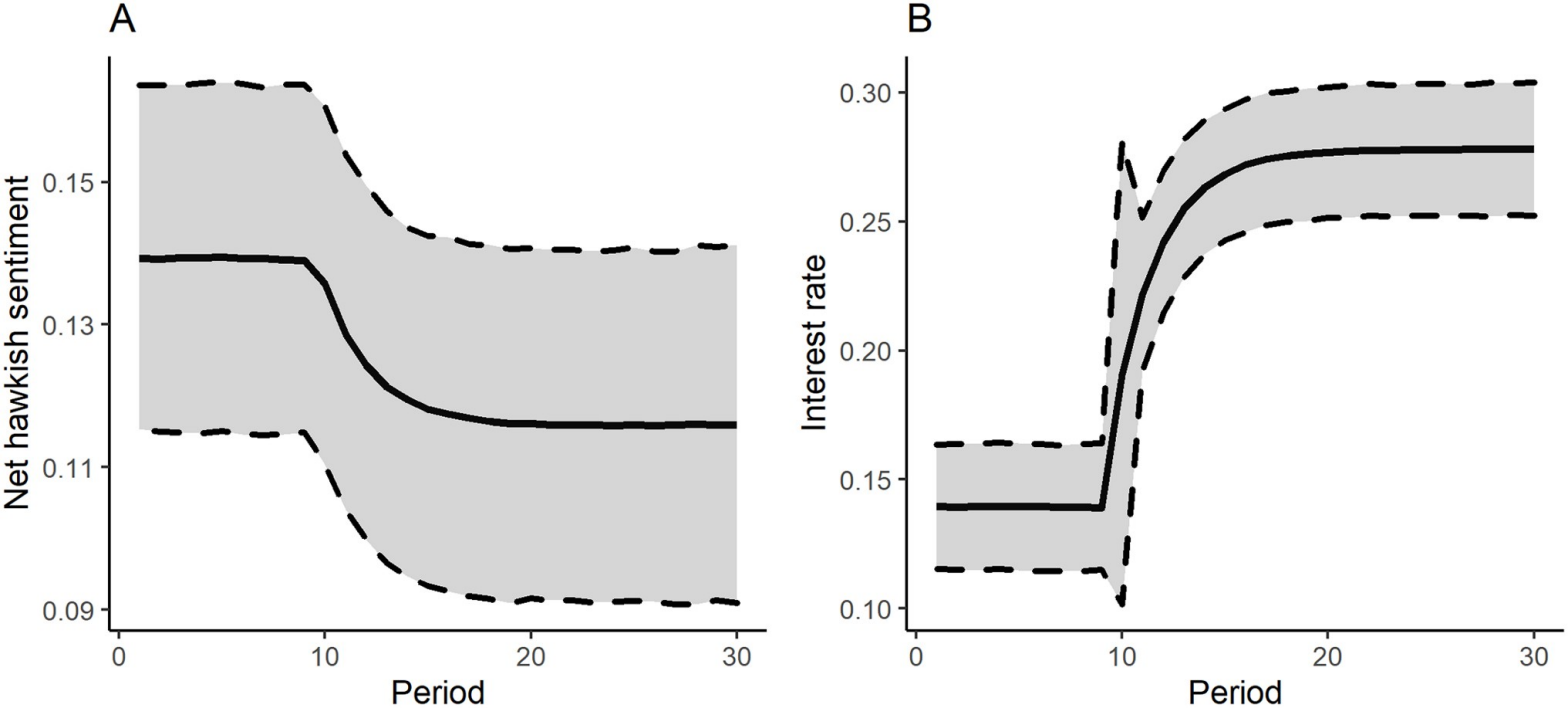
Shock responses (sentiment score and interest rate).

Fig 3 presents the simulated shock responses of the Yield_intra year_ time series due to a shock of a standard deviation in the interest rate (Panel B) and to a shock of the same volume in the nethawkish sentiment variable (Panel A) in the 10th period.

In both cases the responses during the simulation were significant on a 95% error bound. After 30 periods, the shock in the interest rate decreased the mean value of the short run yield factor scores from 0.139 to 0.116, which can be interpreted as a 16,5% decrease. In parallel with that, an interest rate shock increased yield scores up to 0.277 on a same time horizon, which is a huge 97.8% increase. Comparing the effects, it is visible that interest rates have a more profound effect as it is expected from theory. Models for Yield_long term_, and Yield_1-3y_ dependent variable resulted in positive effects of the nethawkish shock which in counterintuitive and highlights the previously discussed problem of potential model misspecification (Please see the other shock responses in the Appendix A).

We also estimated the restricted error correction version (RECM) for our well-functioning short run equation to estimate the error correction term (ECT). Parameter estimation results of course were the same but the RECM version’s ECT parameter aggregated the effect of the lagged level variables. The parameter value of -0.356 (0.071) was significant on a 1% significance level indicating that in short run disequilibrium the time series jointly correct for approximately 36% of the deviations from the long run stochastic equilibrium in each period.

## Conclusion

In this article we investigated how the public communication of the Hungarian Central Bank’s Monetary Council (MC) affects Hungarian sovereign bond yields. Our results showed that central bank forward guidance has an intra-year effect on bond yields. For a shock of one standard deviation in the sentiment variable the intra-year yield variable reacted with a drop of -16.5 per cent after 30 periods. This means that Hypothesis 2 is not supported since we can in fact discern an intra-year effect due to forward guidance operationalized as the hawkish or dovish sentiment of press releases. As a consequence, our empirical results prompt us to revisit the underlying theory of how short-term bond yields are priced and the rationale of forward guidance as a tool aimed at influencing the yield curve over the medium- and long-term. As far as Hypothesis 1 is concerned, the policy rate is still the key explanatory variable when it comes to the yield curve and especially over the medium to long term (along with the central bank governor dummy in the case of the Matolcsy period and the spillover of the FED rate in our OLS-based robustness checks). This finding highlights the interrelated nature of interest rate policy and forward guidance (see Hypothesis 1). Hypothesis 3 is not supported since the sentiment variable was not significantly cointegrated with the time series of bond yields beyond intra-year yields, while our evidence lent support for Hypothesis 4 as the effect forward guidance dissipated for maturities longer than one year.

In sum, our results for well specified autoregressive distributed lag (ARDL) models—having normally distributed and autocorrelation free residuals—suggest that sentiment scores of the policy council press releases are capable of driving the yield curve of government bonds with short term maturity. Cointegration between the intra-year yields, the policy interest rate and the hawkish sentiment content of the minutes was identified with Pesaran and Johansen tests. We also ran robustness checks to situate our models in the context of various control variables. The traditional OLS estimation with Newey-West robust standard errors could only detect the effect of the policy rate as well as spillover effects from the FED and ECB rates and the effect of the governor’s period of András Simor. Furthermore, we checked the robustness of our results by applying different textual variables in our ARDL models, and found that all alternative models support our results.

A further interesting result of our analysis is that the advent of the Matolcsy era (in the form of external members selected by Orbán’s party before Matolcsy was appointed in 2013) serves as a veritable cut-off point for our econometric analysis as well. By 2012 the four Matolcsy-aligned external members formed a majority on the Monetary Council and voting records show that they did not vote for rate increases under any circumstances that year, just as the remaining members of the previous liberal majority did not vote for rate reductions (For a more detailed account of the political context of the MNB, see [55]). As we discussed above we could only make valid statistical statements about the models that were estimated on the last subsample which coincides with the de facto Matolcsy era. Since the structural break almost perfectly coincides with the political takeover in the Hungarian National Bank, dividing the full sample is justified from a statistical, and an economic policy perspective alike. Moreover, in Appendix E we represent our cohesion index, which indicates the accordance monetary council votes in a historical perspective. It is visible that the measure constantly increased during the Matolcsy era, when prior council members were replaced. In sum, our findings show that forward guidance in the form of press release sentiment was effective on intra-year yields in the Matolcsy period, but not for previous periods.

Our research offers a three-fold contribution to the literature. First, from the standpoint of methodology, we apply a quantitative text analysis framework to a case for which similar studies are not available. We compiled a dictionary explicitly tailored to the domain of monetary policy (as opposed to working with general purpose, or even financial, keywords). The dictionary is generalizable to other languages by virtue of the universal lexicon of macroeconomics. Furthermore, we have prepared a new datasets for MNB press releases freely available for further studies of Hungarian monetary policy.

Second, in terms of the scope of application of these methods, we broadened the discussion related to the effectiveness of forward guidance by analyzing a non-eurozone EU country in Central-Eastern Europe. Third, we offer new evidence based on a research design that is independent from the empirical strategy of studies which focus on quantitative, as opposed to qualitative, forward guidance. Still, our time series analysis ties our research directly to mainstream models of the yield curve.

Future work could be aimed at fortifying the internal validity of the analysis, as well as extending its external validity. Dictionary-based methods are appropriate for the limited and recurring vocabulary of central bank minutes and press releases. However, they may be sensitive to paradigm changes in monetary policy (such as the introduction of inflation targeting in the early 2000s) and, in general, the periodic differences of longer time series of press releases (see e.g. format changes). Even with keeping with the dictionary paradigm the list of valence shifters could be extended with diminishers and intensifiers for better results [56].

Supervised learning methods could prove to be a viable alternative to the lexicon-based approach. Machine learning could also be utilized in calculating alternative measures of monetary policy preferences, such as the topic emphasis variable of Baerg and Lowe [43]. A larger than average share of terms related to inflation (such as energy prices or “expectations”) in a policy statement may be indicative of more hawkish sentiment.

From a different angle, interviews with a more comprehensive sample of policymakers, past and present, may shed light on the intricacies and specific role of forward guidance in the general monetary policy toolbox. Qualitative data would be also useful in properly gauging the incentives of bond traders in pricing qualitative central bank communication. Finally, the external validity of our research design should be tested by replicating the analysis for other jurisdictions, starting, perhaps, with the Visegrad countries of Central-Eastern Europe.

## Appendix A—Model diagnostics for the ARDL models

**Fig 4:**
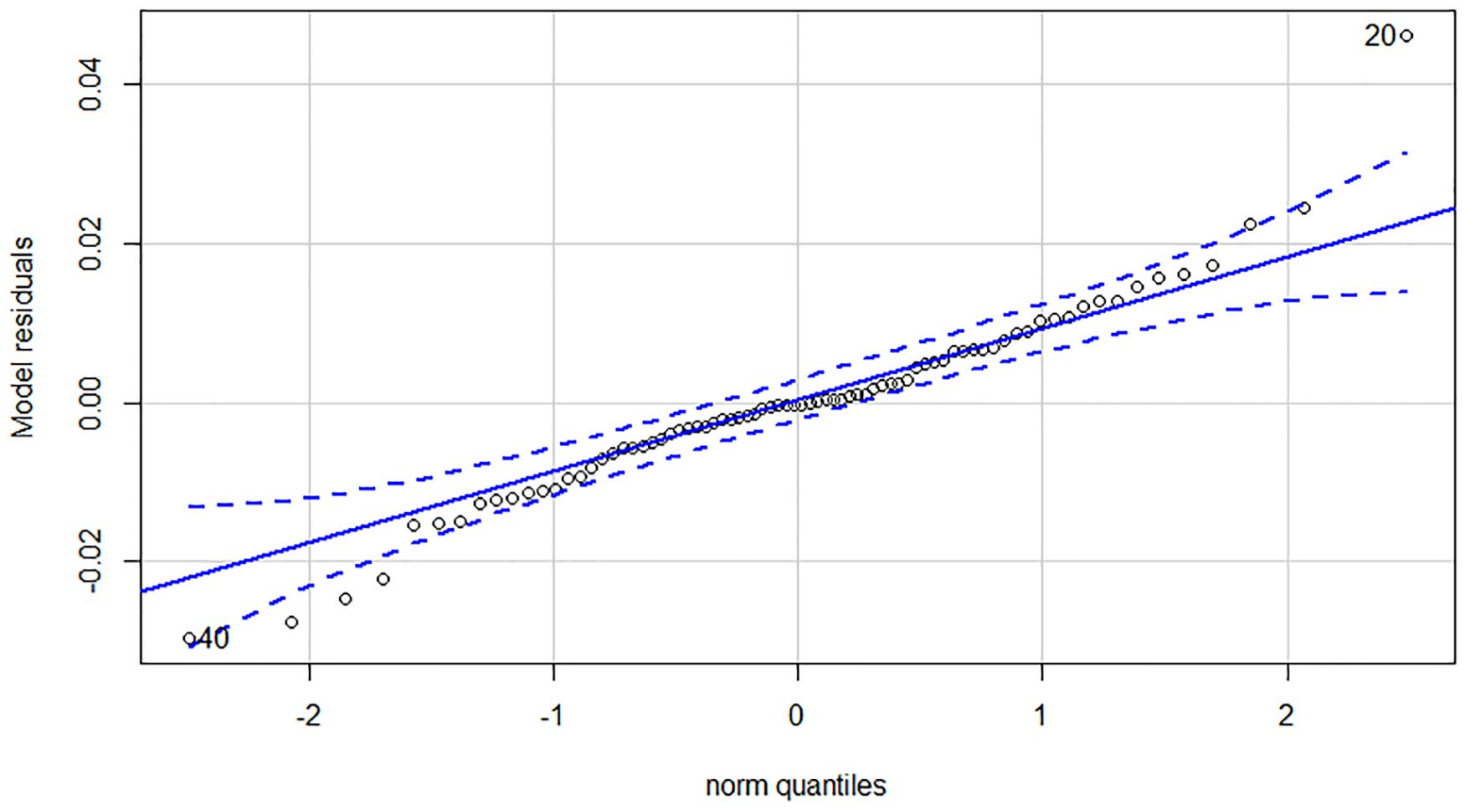
Q-Q plot of the intra year yield model (90-168 subsample).

**Fig 5:**
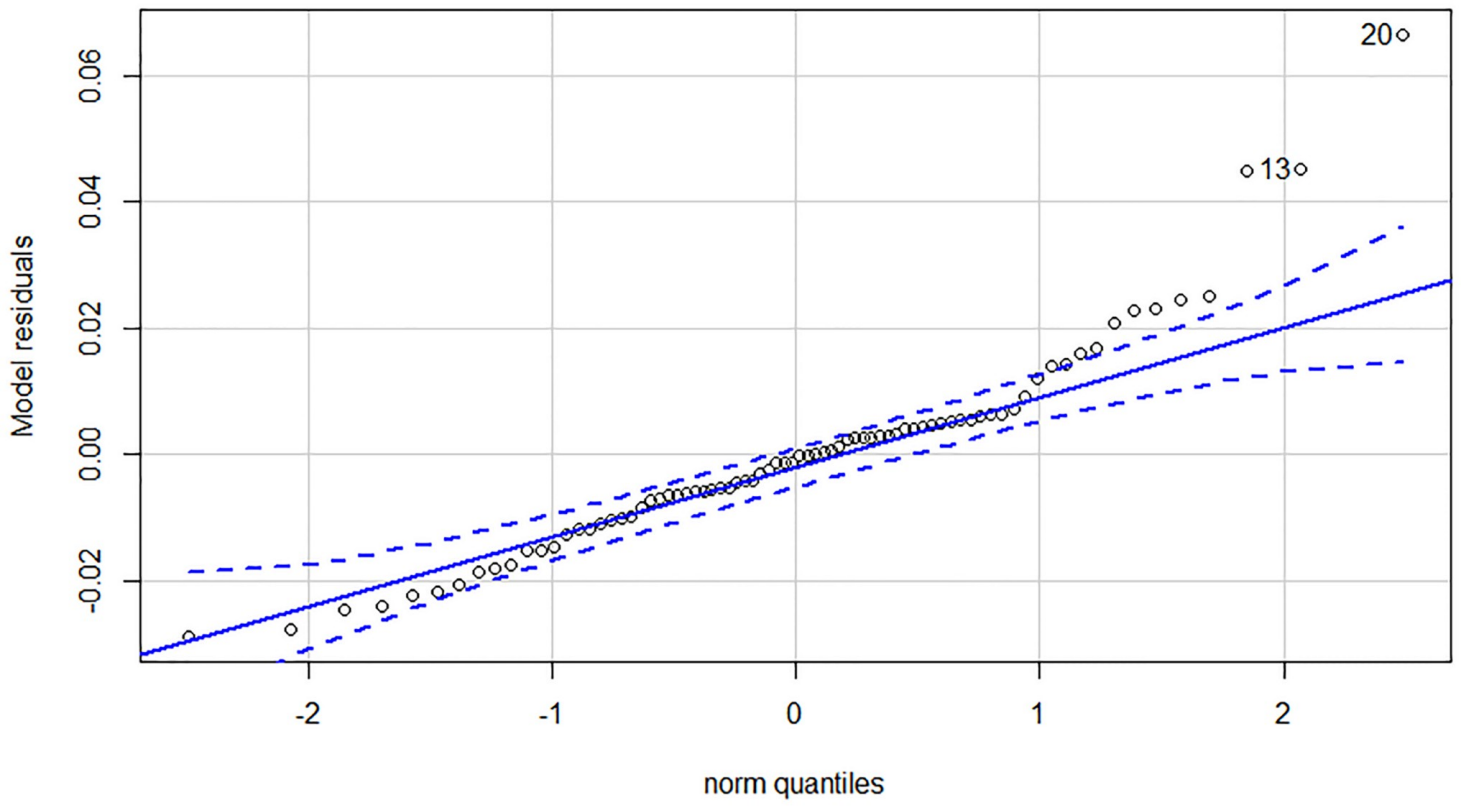
Q-Q plot of the 1 to 3 years yield model (90-168 subsample).

**Fig 6:**
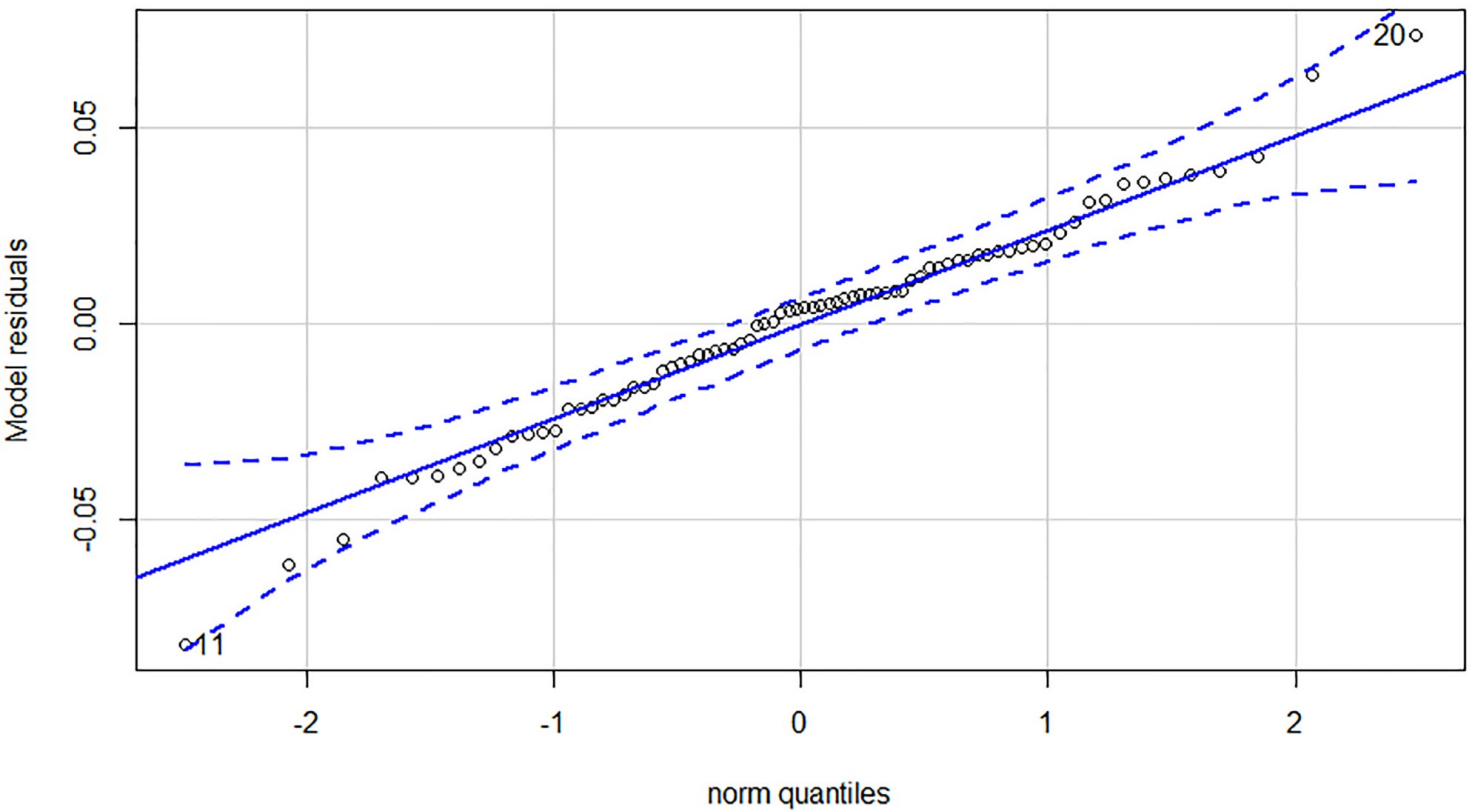
Q-Q plot of the long term yield model (90-168 subsample).

**Fig 7:**
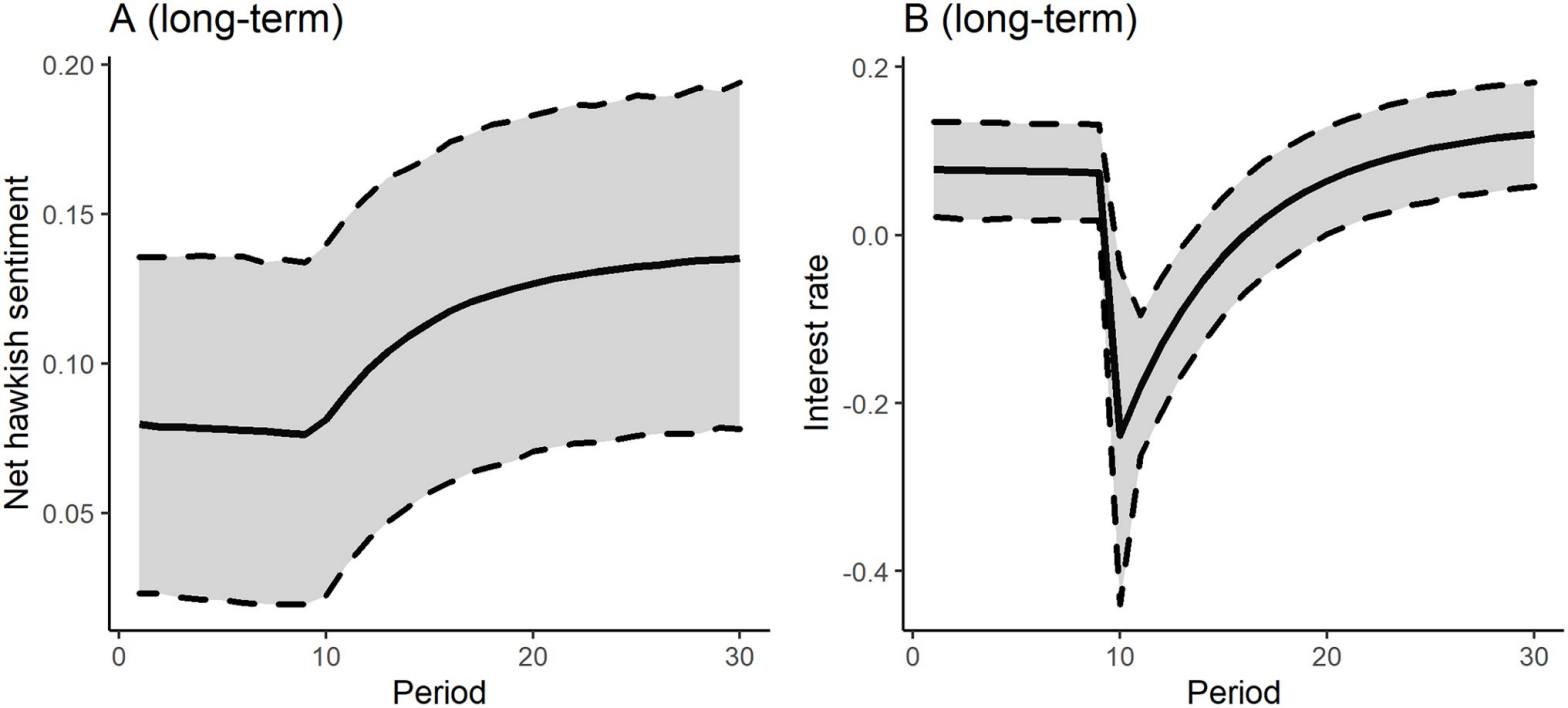
Shock response, long term yield model (90-168 subsample).

**Fig 8:**
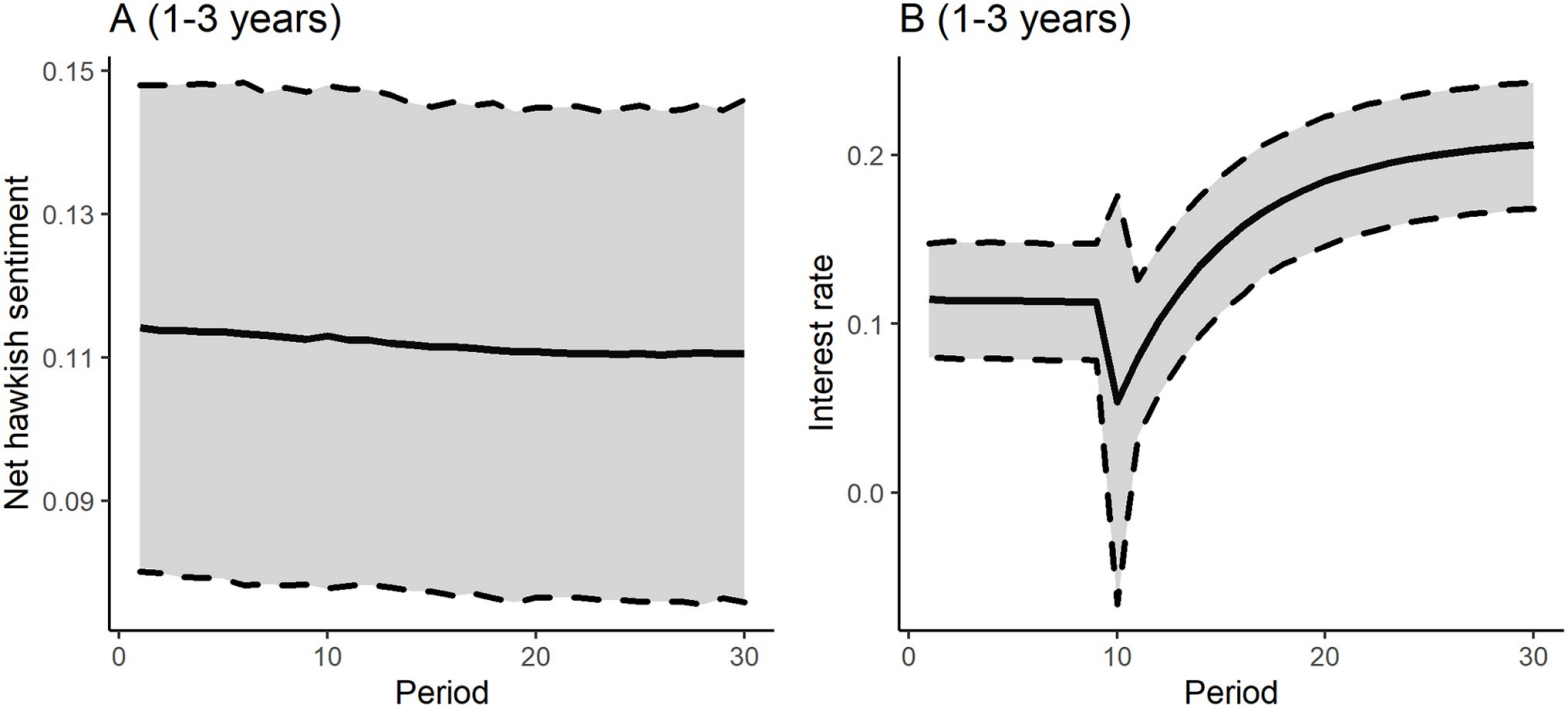
Shock response, 1 to 3 years yield model (90-168 subsample).

In Appendix A we collected the diagnostics of the ARDL models estimated in the article. This includes the Johansen trace test results, the Q-Q plot of the residuals and the shock response plots for the long and medium term models (Table 6, Figs 4–8).

**Table 6:** Johansen trace test results for different dependent variables on the subsample ranging from June 2012 to December 2018.

H0	Test statistic	10%	5%	1%
Yield_long term_ + i + nethawkish				
*r* < = 2	6.15	6.5	8.18	11.65
*r* < = 1	17.26	15.66	17.95	23.52
*r* = 0	70.69	28.71	31.52	37.22
Yield_long term_ + i				
*r* < = 1	10.61	6.5	8.18	11.65
*r* = 0	55.42	15.66	17.95	23.52
Yield_long term_ + nethawkish				
*r* < = 1	5.58	6.5	8.18	11.65
*r* = 0	12.36	15.66	17.95	23.52
Yield_1-3y_ + i + nethawkish				
*r* < = 2	4.36	6.5	8.18	11.65
*r* < = 1	19.37	15.66	17.95	23.52
*r* = 0	64.66	28.71	31.52	37.22
Yield_1-3y_ + i				
*r* < = 1	9.75	6.5	8.18	11.65
*r* = 0	51.85	15.66	17.95	23.52
Yield_1-3y_ + nethawkish				
*r* < = 1	6.95	6.5	8.18	11.65
*r* = 0	20.53	15.66	17.95	23.52
Yield_intra year_ + i + nethawkish				
*r* < = 2	3.84	6.5	8.18	11.65
*r* < = 1	26.37	15.66	17.95	23.52
*r* = 0	70.73	28.71	31.52	37.22
Yield_intra year_ + i				
*r* < = 1	8.02	6.5	8.18	11.65
*r* = 0	50.99	15.66	17.95	23.52
Yield_intra year_ + nethawkish				
*r* < = 1	5.87	6.5	8.18	11.65
*r* = 0	27.26	15.66	17.95	23.52

## Appendix B—Robustness check with alternative textual variables

In this Appendix we present our best performing model using sentiment scores computed with alternative dictionaries, such as the one by Loughran and McDonald as well as that of Apel, Grimaldi and Hull. Each of these models show a similar level and direction of cointegration (with a higher level of significance for our own dictionary) which underpins our detailed results for our own dictionary presented in the Results section (Table 7).

**Table 7:** Robustness test result using sentiment scores computed with alternative dictionaries.

	Yield_intra year_		
Sample	90-168		
Dictionary	nethawkish	Loughran-McDonald	AGH
	Model 9	Model 10	Model 11
*Intercept*	0.008	0.626*	0.025
	0.008	(0.302)	(0.018)
*dependent*_*t*−1_	-0.356***	-0.309***	-0.250***
	(0.0758)	(0.079)	(0.063)
*i*_*t*−1_	0.028***	0.025***	0.020***
	(0.006)	(0.079)	(0.063)
*sentiment*_*t*−1_	-0.015**	-0.644*	-0.034*
	(0.005)	(0.306)	(0.016)
Δ*i*	0.029 (0.024)	0.026	0.031
	(0.024)	(0.024)	(0.024)
Δ*sentiment*	-0.006 (0.006)	-0.926**	0.023
	(0.006)	(0.284)	(0.018)
R-squared	0.427	0.443	0.393
R-squared	0.387	0.404	0.433
ECT	-0.356***	-0.309***	-0.250***
	(0.071)	(0.074)	(0.056)
BP p-value	0.919	0.894	0.947
Coint.	1%	5%	1%

## Appendix C—Alternative model specification and estimation

To test our hypothesis we used a Newey-West estimation which provides heteroscedasticity and autocorrelation consistent standard errors and addresses the violations of the Gauss-Markov conditions which are not met when using a simple OLS method. As a follow-up, we also tested for cointegration between the bond yields and (i) interest rate, and (ii) the sentiment score using autoregressive dustributed lag models. We estimated an equation which, after controlling for other factors, allows us we investigate whether sentiment scores have a direct effect on the term structure without the transmission of the policy rate. In the model, we differenced all non-stationary variables. To make interpretation more natural, we took the logarithm of our dependent variable before differencing; thus, after the transformation, the dependent variable has a percentage interpretation when changes are moderate.

Eq 3 displays our first model specification:

$$\begin{array}{c} \log\left({\text{Yield}}_{\text{kt}}\right)=\alpha_{0}+\alpha_{1}\log\left({\text{nethawkish}}_{t}\right)+\sum_{i=2}^{n}\alpha_{i}\Delta {\text{Control}}_{it}+\sum_{j=n+1}^{m}\alpha_{j}\Delta {\text{Control}}_{jt}+\epsilon_{t} \end{array}$$ (3)

where *Yield*_*kt*_ denotes the value of the yield curve of a specific k composite term length corresponding to our relevant time frames (i.e. Yield_intra year_, one-to-three year or long), at time t. This dependent variable is explained by nethawkish_t, the actual value of the sentiment scores at time t. We separated our control variables into non-stationary and stationary groups. The third term on the right-hand side of Eq (1) lists the differenced non-stationary variables, while the fourth term continues with the stationary money supply, dummy variables for MNB governor Simor and Matolcsy, complemented with and Monetary Council voting cohesion index (mt_cohesion).

Eq 4 extends the baseline model with the change of interest rate as a direct effect on yield:

$$\begin{array}{c} \log\left({\text{Yield}}_{\text{kt}}\right)=\beta_{0}+\beta_{1}\Delta {\text{IR}}_{t}+\beta_{2}\log\left({\text{nethawkish}}_{t}\right)+\sum_{i=3}^{n}\beta_{i}\Delta {\text{Control}}_{it}+\sum_{j=n+1}^{m}\beta_{j}\Delta {\text{Control}}_{jt}+\epsilon_{t} \end{array}$$ (4)

This specification includes the non-stationary policy variable in a differenced form, to eliminate the potential spurious relationship caused by a common trend in the two time series. Moreover, the model separates the direct effect of a policy change from the collateral effect of the sentiment behind the decision.

### Control variables for the Newey-West estimation

Since government bond yields and the features of the yield curve are one of pivotal indicators for monetary policy, a vast amount of research was consecrated to understanding the variables which have on effect on them. Models are built up from the most important policy instrument in the monetary policy toolkit, the policy rate (see also monetary policy “instrument” or “stance”; The time series for the relevant policy variable, the “key interest rate” (i), was taken from the MNB data repository (see Table 8). We gathered monthly data, for variables where it is applicable, and except when noted otherwise, from January 2005 to December of 2018.

**Table 8:** Control variables and data sources.

Control factor	In models	Source
Interest rate	I	MNB Monetary policy instruments
Unemployment	Unemployment rate	MNB Main macro statistics
M3	m3	MNB Main macro statistics
Core inflation	Core consumer price index	MNB Price statistics
HUF/USD	USD	Eurostat
HUF/EUR	EUR	Eurostat
MNB Governor dummy	Simor, Matolcsy	MNB
Council cohesion	Council cohesion	MNB, authors’ calculations
FED rate	FED rate	FRED (St. Louis FED)
ECB rate	ECB rate	ECB

This latter served as the source for multiple other control variables, which are considered to be major drivers of the term structure of interest rates (as used in the literature listed in Table 8). These staples of bond yield models include the three-month average of the unemployment rate (u), the monetary supply measure (m3), one of the widely used for inflation forecasting and the year-on-year core inflation (core_i_yoy) figure [57]. As further controls related to exchange rates, necessary for the study of a small open economy such as Hungary, the monthly average Hungarian Forint exchange rate of US dollar (USD) and euro (EUR) were collected from Eurostat. We added three variables to our model which put monetary policy in a political context. The cohesion index of the monetary council is computed based on voting records published by MNB (for more information and descriptive statistics, see Appendix E). This approach is also used to predict rate changes and termed as skew [58, 59]. Our variable captures a similar effect, although the calculation is slightly different. The cohesion variable also reflects the finding of Ainsley [60] who showed how the rate preference of the monetary council under Governor Matolcsy is lower than the councilmembers under the tenure of his predecessors. In order to capture the conflicting economic policy ideologies of subsequent governor periods, we also introduced two dummy variables. The base period is the term of Zsigmond Járai (appointed by a right-wing government, and serving from 2001 to 2007), and the values refer to the period of András Simor (appointed by a left-liberal coalition, serving from 2007 to 2013) and that of György Matolcsy (appointed by a right-wing government in 2013; we only used data for this period up to 2018).

The literature that investigates monetary policy outside of the Euro area provides important insights for control variables to account for the small open economy policymaking environment of the Hungarian Central Bank [61, 62]. These include the policy rates and balance sheets of both the Federal Reserve and the European Central Banks. The inclusion of these variables are an important step to capture the market moving capabilities of the secular decrease of interest rates and the unconventional monetary policy tools (which are reflected by the balance sheets).

### Results of the Newey-West estimation

We conducted regression analysis on our dataset in order to gauge the effect of the sentiment of central bank press releases on the yield curve after controlling for other frequently deployed variables. Therefore, we estimated our ordinary least squares models with Newey-West robust standard errors.

The estimation results for the benchmark models with no textual variable is in Table 9. The key policy rate has the expected sign and is statistically significant in all models. When the models do not include the policy rate variable then only the Matolcsy governor dummy is significant in the short term. In a substantive interpretation, the result for the model with 1-to-3 year yield as the dependent variable indicates that in a hypothetical case where the starting average return rate of different government bonds is around 5 percentage points, a 3.6 percentage point increase in the interest rate moves the yield up by around 1 percentage point (a 22 percent increase). In line with the literature we also find that the FED and ECB policy rate is also significantly associated with the intra year and long term yield changes.

**Table 9:** Newey-West estimation results.

	Intra-year yield		1-to-3-year yield		Long-term yield	
	without policy rate	incl. policy rate	without policy rate	incl. policy rate	without policy rate	incl. policy rate
ln(Nethawkish)	−0.007	−0.009	0.003	0.001	−0.000	−0.003
	(0.014)	(0.013)	(0.013)	(0.012)	(0.017)	(0.015)
Policy rate		0.105***		0.065*		0.097***
		(0.023)		(0.030)		(0.018)
Unemployment rate	0.073	0.084	−0.020	−0.013	−0.000	0.010
	(0.056)	(0.055)	(0.052)	(0.054)	(0.041)	(0.044)
M3	−0.006	−0.006	0.003	0.003	−0.003	−0.003
	(0.005)	(0.005)	(0.004)	(0.004)	(0.005)	(0.005)
Core inflation	−0.069	−0.072	−0.023	−0.024	−0.088	−0.090
	(0.038)	(0.040)	(0.030)	(0.029)	(0.062)	(0.063)
ECB rate	0.057	0.103**	−0.031	−0.002	0.059	0.102**
	(0.043)	(0.035)	(0.060)	(0.062)	(0.040)	(0.038)
FED rate	−0.189	−0.116	−0.038	0.007	−0.162***	−0.094
	(0.109)	(0.121)	(0.100)	(0.117)	(0.044)	(0.057)
Simor	−0.118*	−0.086	−0.005	0.014	−0.088	−0.059
	(0.058)	(0.060)	(0.047)	(0.051)	(0.063)	(0.065)
Matolcsy	−0.127	−0.097	−0.030	−0.011	−0.095	−0.067
	(0.069)	(0.073)	(0.056)	(0.060)	(0.080)	(0.083)
Council cohesion	0.048	0.035	0.023	0.015	0.037	0.025
	(0.063)	(0.049)	(0.062)	(0.055)	(0.064)	(0.055)
R^2^	0.040	0.050	0.008	0.011	0.021	0.029
Adj. R^2^	−0.017	−0.015	−0.052	−0.056	−0.038	−0.036
Num. obs.	159	159	159	159	159	159

*** *p* < 0.001,

** *p* < 0.01,

* *p* < 0.05, standard errors in parenthesis.

Table 9 also presents estimation results for the model augmented with the textual variable. The ln(Nethawkish) sentiment variable has no discernible effect on the yield curve. This means that the expectation of interest rate increases due to a hawkish sentiment in the press releases does not urge investors to ramp up demand for no-risk bonds and, therefore, this causal logic does not lead to price increases and lower yields.

## Appendix D—Example dictionary output

In this Appendix we first provide an excerpt from our coded documents (see Table 10). It shows a sample (the first 6 sentences) from the 2011-09-20 press release, the most dovish document in our corpus. The table only shows categories where the dictionary lookup actually found matches.

**Table 10:** Sample dictionary coding of an MNB press release.

Original sentence	Macro normal	Hawk normal	Dove normal	Inflation term	Inflation dove	Inflation hawk
the monetary council discussed and commented on the september issue of the mnbs quarterly report on inflation prepared by bank staff		0	0	*the mnbs quarterly report on inflation prepared by bank staff*	0	0
in the councils judgement hungarian economic growth is likely to remain subdued over the next two years with the level of output remaining below its potential throughout the period	*in the councils judgement hungarian economic growth is likely to remain subdued years with the level of output remaining below its potential throughout*	0.04	0.09		0	0
mediumterm upside risks to inflation have fallen due to weak domestic demand	*fallen due to weak domestic demand*	0	0.33	*mediumterm upside risks to inflation have fallen due to weak*	0.2	0
*inflation may fall back to by the beginning of as the effects of cost shocks and increases in indirect taxes wear off*		0	0	*inflation may fall back to by*	0.17	0
the monetary council is closely monitoring developments in taxadjusted core inflation in addition to movements in the consumer price index		0	0	*monitoring developments in taxadjusted core inflation in addition to movements in the consumer price index*	0	0

Note: Excerpt (the first 6 sentences) from the 2011-09-20 press release. The table only shows categories where the dictionary lookup actually found matches.

## Appendix E—The monetary policy council cohesion variable

**Fig 9:**
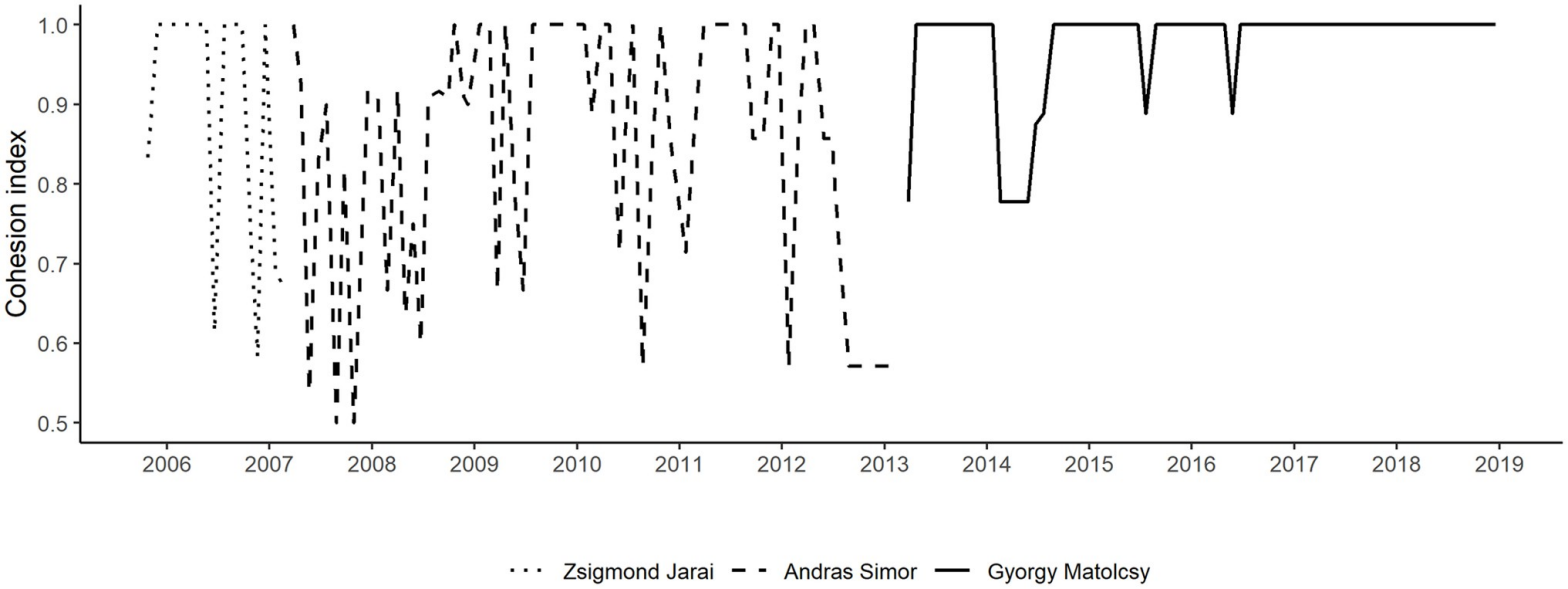
The time series for the MPC cohesion variable.

The cohesion index of the monetary council is computed based on voting records published by MNB. The index values reflect how divided the monetary council was for each rate decision session based on how many of its members voted in the same direction. When all the members vote for the same decision (all hold, all increase or all decrease), the index will be 1.0. In the event of an equal split in the votes the index will be 0.5. In all other cases the index is computed as 1—(sum of votes / number of members). The change in the governance of the national bank is reflected in our cohesion index and, as Fig 9 demonstrates, under Governor Matolcsy, dissent in rate decision voting decreased substantially. The official data covers the rate decisions from October 24, 2005 onwards (with the exception of the meeting in October 22, 2008, which was an unscheduled rate decision at the height of the financial crisis which is still not public).
